# Isolated Late Metastasis of a Renal Cell Cancer Treated by Radical Distal Pancreatectomy

**DOI:** 10.1155/1996/56065

**Published:** 1996

**Authors:** J. P. Barras, H. Baer, A. Stenzl, A. Czerniak

**Affiliations:** 1 University clinic for visceral and transplantation surgery Inselspital Bern Bern CH-3010 Switzerland; 2 Department of urology University of Bern Inselspital Bern CH-3010 Switzerland

## Abstract

A 53–year-old man underwent right nephrectomy for a locally advanced renal cell carcinoma with concomitant resection of a solitary metastasis in the right lung. Ten years later, he presented with haematochezia caused by a tumour in the tail of pancreas, invading the transverse colon and the greater curvature of the stomach. The tumour was radically resected, and histological examination revealed a solitary metastasis of the previous renal cell carcinoma. This case illustrates a rare indication for pancreatic resection because of pancreatic metastasis.


*HPB Surgery*, 1996, Vol.10, pp. 51–54
Reprints available directly from the publisher
Photocopying permitted by license only

© 1996 OPA (Overseas Publishers Association)
Amsterdam B.V. Published in The Netherlands
by Harwood Academic Publishers GmbH
Printed in Malaysia

### CASE REPORT
### Isolated Late Metastasis of a Renal Cell Cancer Treated by Radical Distal Pancreatectomy

J.P. BARRAS*, H. BAER*, A. STENZL° and A. CZERNIAK*

*University clinic for visceral and transplantation surgery, Inselspital Bern, CH-3010 Bern, Switzerland
°Department of urology, University of Bern, Inselspital, CH-3010 Bern, Switzerland

*(Received 30 July 1995)*

A 53–year-old man underwent right nephrectomy for a locally advanced renal cell carcinoma with concomitant resection of a solitary metastasis in the right lung. Ten years later, he presented with haematochezia caused by a tumour in the tail of pancreas, invading the transverse colon and the greater curvature of the stomach. The tumour was radically resected, and histological examination revealed a solitary metastasis of the previous renal cell carcinoma. This case illustrates a rare indication for pancreatic resection because of pancreatic metastasis.

KEY WORDS: Metastatic renal cell carcinoma late metastasis pancreatic metastasis pancreatic resection

### INTRODUCTION

At the time of diagnosis approximately 23% of all renal cell carcinomas are metastatic[1]. Most patients present with multiple metastases, solitary metastasis being rare. The value of surgical treatment in such cases, i.e. nephrectomy plus excision of one or more metastases, in uncertain, but it is sometimes carried out for lack of satisfactory alternatives. Solitary late recurrence, defined as metastasis occurring 10 years or more after diagnosis of the primary renal tumour is also uncommon and has an unfavourable prognosis. Pancreatic metastases are much less common than pulmonary metastases but can be the presenting feature of a kidney neoplasm[2].

### CASE REPORT

A 53-year-old man presented in 1982 with a solitary right pulmonary nodule on a routine chest X-ray and a large right flank mass of renal origin on computer tomography (CT). Detailed imaging revealed no other tumours. A right nephrectomy and an inferior right pulmonary lobectomy were performed. The histology showed a well-differentiated clear cell carcinoma penetrating into the perirenal fat (pT3) without lymph node involvement. The 3 cm diameter pulmonary nodule was a well demarcated metastasis. Annual checks revealed no evidence of recurrent disease. In 1992 haematochezia developed, and colonoscopy showed an infiltrating tumour of the left colonic flexure. On abdominal CT (Fig. 1) a large tumour was seen infiltrating the body and tail of pancreas, left colonic flexure and greater curvature of the stomach; chest X-ray and bone scan showed no other lesions. The patient underwent en-bloc tumour excision with distal pancreatectomy, splenectomy, partial gastrectomy and left hemicolectomy. Histologically a metastatic lesion was found consisting of a moderately differentiated clear cell carcinoma. Comparison of this pathology specimen with the original renal tumour revealed an almost identical tumour. The postoperative course was uneventful and the patient was discharged on the tenth postoperative day. At 6 months follow-up, he was well without evidence of recurrence on abdominal or thoracic CT scan.

*Correspondence to*: Dr. med. Jean-Pierre Barras, Universitätsklinik für Viszerale und Transplantationschirurgie, Inselspital, CH-3010 Bern, Switzerland, Phone: 031 64 37 55 Fax: 031 28 47 72

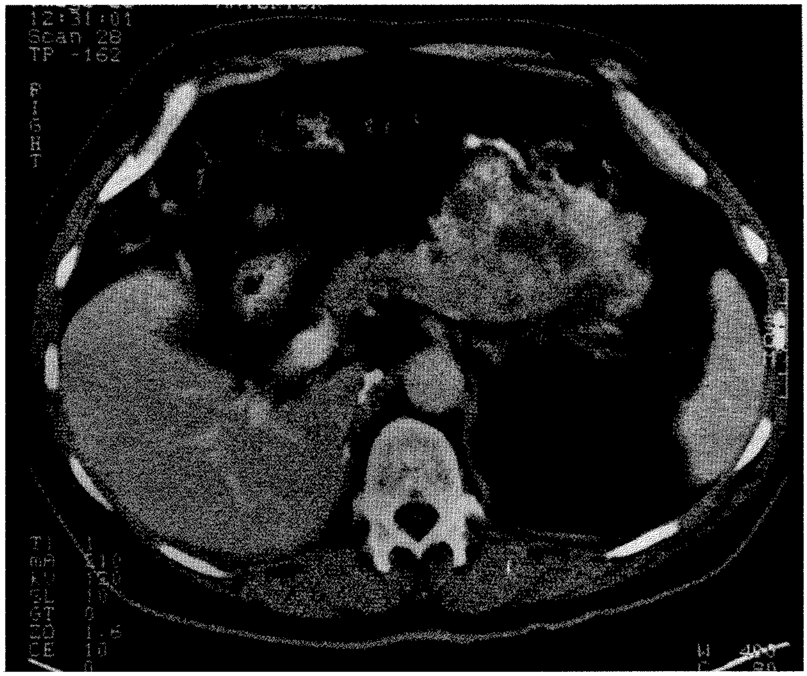

**Figure 1** Abdominal CT in 1992 depicting a tumorous lesion in the tail of the pancreas invading the transverse colon and the greater curvature of the stomach.

### DISCUSSION

Metastatic renal cell carcinoma, whether synchronous or metachronous, has a poor prognosis, five-year survival rates range from 0 to 19%[3], and survival beyond 10 years is rare[4,5]. In selected series, combined resection of the primary renal tumour and of a solitary lung metastasis has produced a 5-year survival rate of 25–35%[6].

Late recurrence of a renal tumour after curative resection is uncommon, but it can occur after disease-free intervals of several years. Petersen found 23 reported cases of recurrent renal carcinoma after 10 to 37 years[3]. Most of these recurrences were in the lung, and recurrence in the pancreas, colon or stomach has not been described. Prolonged survival can follow surgical excision of the late metastasis.

Although our patient presented initially with a synchronous pulmonary metastasis, he did well for 10 years after radical tumour surgery. Again, despite the local extension of the pancreatic metastasis, radical excision was both feasible and well tolerated by the patient. Abnormal tumour-host interactions may be responsible for the unusual sites and intervals of recurrence observed. Removal of a solitary late metastasis can be followed by further disease-free survival of up to 17 years[7].

Operative treatment is seldom indicated for pancreatic metastases although there are some reports for isolated metastasis of melanoma[8]. Seventeen cases of renal carcinoma presenting with pancreatic metastasis have been reported[2,9,10,11], and this can be an occasional indication for pancreatectomy.

### REFERENCES

1. Stenzl A. deKernion J.B. (1989) Pathology, biology and clinical staging of renal cell carcinoma. *Sem in Oncology* **16** (Suppl 1), 3–11.
2. Marquant J. Giraud B. Maliakas S. (1971) Pancreatic metastasis revealing a kidney neoplasm. *J Urol Nephrol (Paris)* **77** (7), 595–601.
3. Petersen R.O. (1986) *Urologic Pathology*, pp 57–179, Philadelphia, Lippincott.
4. McNichols D.W. Segura J.W. DeWeerd J.H. (1981) Renal cell carcinoma: long term survival and late recurrence. *J. Urol* **126** (1), 17–23.
5. Pritchett T.R. Lieskovsky G. Skinner D.G. (1988) Clinical manifestations and treatment of renal parenchymal tumors. In Skinner D.G. Lieskovsky G, (eds): *Diagnosis and management of genotourinary cancer.* Philadelphia, pp 337–61, WB Saunders Co,
6. deKernion J.B (1983) Treatment of advanced renal cell carcinoma-traditional methods and innovative approaches. *J. Urol* **130**, 2–7.
7. Middleton R.G. (1967) Surgery for metastatic renal cell carcinoma. *J Urol* **97**, 973–6.

8. Strauch S. Rosch W. (1989) Obstructive jaundice caused by metastasis to the head of the pancreas by a malignant melanoma. *Bildgebung* **56** (3), 115–7.
9. Py J. M. Arnaud J. P. Cinqualbre J. Adolff M. Bollack C. (1984) Pancreatic metastases of nephro-epitheliomas. Apropos of 2 cases. *Acta Chir Belg.* **84** (3), 117–21.
10. Gohju K. Mizuno Y. Gotoh A. Takena A. Imai T. Kamidono S. Yamanaka N. Harada K. (1990) A clinicopathological analysis of renal cell carcinoma in solitary kidney. *Nippon Gan Chiryo Gakkai Shi.* **25** (5), 965–971.
11. Oka H. Hatamyama T, Taki Y. Ueyama H. Hida S. Noguchi M. (1991) [A resected case of renal cell carcinoma with metastasis to pancreas] *Hinyokika Kiyo* **37**, 1531-4.

### *Invited Commentary*

Metastasis to the pancreas is distinctly uncommon, being described at autopsy in only 3-5% of patients with generalized malignancies[1,2]. Usually the primary tumour is bronchogenic carcinoma, breast carcinoma or melanoma, but metastases have been reported from a wide variety of primaries such as the thyroid, colon and kidney[3,4].

Renal cell carcinoma, in particular, can behave in a variable and somewhat unpredictable manner. Although most patients with this disease die within a year, up to 20%[5] may have periods of slow tumour growth or stability lasting many years[6], during which time metachronous carcinoma or metastasis to unusual sites may occur despite radical resection of the initial tumour[7]. In a review of 506 patients with renal cell carcinoma, Mc Nichols *et al.* found that among the 158 patients who survived more than 10 years, 11% had late recurrence in the form of metastases[8]. As with the case of Dr. Barras, 25% of all renal cell carcinoma patients first present with metastasis[9, 10], usually to the lungs, lymph nodes, liver and bone. The site of metastasis seems to influence survival and response to treatment. In a study of 181 cases, Maldazys *et al.*[5] noted that patients with metastasis limited to the lung parenchyma survived longer than those with metastasis to other single or multiple organs.

Metastatic involvement of the pancreas is often part of widespread nodal and visceral involvement[11], while solitary metastasis to the pancreas is rare, with an incidence of 1–3%[12]. Metastasis to the ampulla of Vater is even more uncommon; very few cases have been reported so far, as shown by Tsao *et al.* in the present report. Most of these patients presented with symptoms of gastrointestinal bleeding and/or jaundice, whereas half those with pancreatic metastasis may be asymptomatic[6]. The patient of Dr. Barras who presented with haematochezia secondary to colonic erosion was unusual.

The five-year survival rate of patients with untreated metastatic renal cell carcinoma is poor, usually less than 13%[13]. With surgical removal of pulmonary metastases, Tolia and Whitmore[12] noted a 35 % five-year survival rate. Because of the small number of cases, no clear conclusions can be drawn regarding pancreatic metastasis from renal cell carcinoma. However, a recent review of the world literature covering sixteen patients with resection of renal metastases to the pancreas endorsed the value of operative treatment[14]. In general, the factors associated with a favourable prognosis after resection of such metastases include: 1) A long interval from the time of resection of the primary tumour, 2) evidence of a solitary lesion on radiographic examination, even if multiple nodules are found at operation, 3) demonstration of extensive necrosis in the resected specimen[5,14]. The extent of pancreatectomy should be dictated by the location of the lesion and the need for an adequate margin of resection. If the disease is limited to the pancreas (or ampulla) or even if it is locally spreading but technically resectable (as in the patient of Dr. Barras), a radical surgical strategy is justified. It is not clear why Dr. Tsao and her colleagues elected to perform a transduodenal excision of the ampullary metastasis in their second patient rather than a formal pancreatoduodenectomy which might conceivably have prevented local recurrence.

As adjuvant irradiation and/or chemotherapy does not seem to prolong the survival of patients with pancreatic metastases, including renal secondaries[3], and because the outlook is generally dismal, it seems that radical surgery should be offered to these rare patients.

G. A. NAWFAL
RCN WILLIAMSON
Department of Surgery
Royal Postgraduate Medical School
Hammersmith Hospital
London, United Kingdom

### REFERENCES

1. Willis R.A. (1973) The spread of tumors in the human body.Third edition. Butterworths, pp 216–217.
2. Whittington R. Moylan D. G. Dobelbower R. R. Kramer S. (1982) Pancreatic tumors in patients with previous malignancy. *Clin. Radiol.* **33**: 297–9.
3. Roland C. F. Van Heerden J. A. (1989) Non pancreatic primary tumors with metastasis to the pancreas. *Surg. Gynecol. Obstet.* Apr; **168** (4): 345–7.

4. Charnsangavej C, Whitley N. O. (1993) Metastases to the pancreas and peri-pancreatic lymph nodes from carcinoma of the right side of the colon: CT. findings in 12 patients. *Am. J. Roentgenol.* Jan; **160** (1): 49–52.
5. Maldazys J. D. De Kernion J. B. (1986) Prognostic factors in metastatic renal carcinoma. *The Journal of Urology* **136**: 376–379.
6. Rypends F. Van Gansbeke D. Lambilliotte J. P. Van Regemorter G. Verhest A. Struyven J. (1992) Pancreatic metastasis from renal cell carcinoma. *The British Journal of Radiology* **65**: 547–548.
7. Kradjian R. Bennington J. (1965) Renal carcinoma recurrent 31 years after nephrectomy. *Arch. Surg.* **90**: 192–5.
8. Mc Nichols D. Segura J. De Weerd J. (1981) Renal cell carcinoma in long-term survival and late recurrence. *J. Urol.* **126:** 17–23.
9. Richie A. W. Chisholm G. D. (1983) The natural history of renal carcinoma. *Sem. Oncol.* **10**: 390–400.
10. Woodhouse C. R. J Adenocarcinoma of the kidney: Review of treatment options. Urological oncology: Dilemmas and development. Wiley. 1991.
11. Strijk S. ( 1989) Pancreatic metastases of renal cell carcinoma: report of two cases. *Gastrointestinal Radiology* **14**: 123–126.
12. Tolia B. Whitmore W. (1975) Solitary metastasis from renal cell carcinoma. *The Journal of Urology* **114**: 836–8.
13. De Kernion J. Rammings K. Smith R. (1978) The natural history of metastatic renal cell carcinoma: A Computer Analysis *The journal of Urology*; **120**: 148–52.
14. Stankard C. E. Karl R. C. (1992) The treatment of isolated pancreatic metastases from renal cell carcinoma: A surgical review. *American Journal of Gastroenterology* **87** (11): 1658–60.

